# Lumbosacral Transitional Vertebrae Influence on Acetabular Orientation and Pelvic Tilt

**DOI:** 10.3390/jcm11175153

**Published:** 2022-08-31

**Authors:** Luis Becker, Nima Taheri, Henryk Haffer, Maximilian Muellner, Christian Hipfl, Katharina Ziegeler, Torsten Diekhoff, Matthias Pumberger

**Affiliations:** 1 Center for Musculoskeletal Surgery, Charité—University Medicine, Charitéplatz 1, 10117 Berlin, Germany; 2 Berlin Institute of Health, Julius Wolff Institute for Biomechanics and Musculoskeletal Regeneration, Charité—University Medicine, Augustenburger Pl. 1, 13353 Berlin, Germany; 3 Department for Radiology, Charité—University Medicine, Charitéplatz 1, 10117 Berlin, Germany

**Keywords:** LSTV, hip arthroplasty, acetabular version, acetabular inclination, acetabular anteversion

## Abstract

Lumbosacral transitional vertebrae (LSTV) are common congenital variances with a prevalence found in the population up to 35.6%. The literature demonstrates an influence of LSTV on bony pelvic anatomy. The influence on the anatomical acetabular orientation, which is important for cup positioning in total hip arthroplasty, has not yet been described for patients with LSTV. A total of 53 patients with LSTV were identified from a CT Database including 819 subjects. Fifty patients with LSTV could be included and were matched for age and sex against a control group. We examined the influence of LSTV, classified according to Castellvi, on acetabular orientation and pelvic tilt in the supine position. Functional acetabular anteversion and inclination, assessed against the table plane, were compared against anatomical acetabular anteversion and inclination, assessed against the anterior pelvic plane. The anatomical acetabular inclination correlated with the pelvic tilt (r = 0.363, *p* < 0.001). The anatomical acetabular inclination was significantly larger than the functional acetabular inclination in the supine position (*p* < 0.001). Castellvi grading of LSTV correlated negatively with pelvic tilt (ρ = −0.387, *p* = 0.006). Castellvi grading correlated significantly with functional acetabular anteversion (ρ = 0.324, *p* = 0.022) and anatomical acetabular anteversion (ρ = 0.306, *p* = 0.022). A higher Castellvi grading was accompanied by a reduced pelvic tilt in the supine position. The functional acetabular anteversion and anatomical acetabular anteversion increased in parallel to the higher Castellvi grading. Therefore, LSTV and Castellvi grading might be assessed on pre-operative X-rays prior to hip arthroplasty and surgeons might consider adjusting cup positioning accordingly.

## 1. Introduction

Lumbosacral transitional vertebrae (LSTV) are one of the most common congenital variances of the spine [1,2]. The literature reports a prevalence of 5%, up to 35.6%, for LSTV [2,3]. LSTV result from partial or complete sacralization of the last lumbar vertebra or partial or complete lumbarization of the first sacral vertebra [2,4,5]. 

Along with the changes in the lumbosacral junction, patients with LSTV present changes in the anatomy of the bony pelvis [6]. The literature shows that patients with LSTV have a higher riding iliac crest when compared to a matched control group [7]. Additionally, patients with LSTV have a larger pelvic incidence, which is an important parameter for assessing sagittal spinal alignment and, in interaction with lumbar lordosis, largely determines the surgical planning for spinal profile restoration [8,9,10,11,12]. However, pelvic incidence also has an influence on acetabular orientation. Radcliff et al. reported in patients without LSTV an association between an increased pelvic incidence and a more vertical acetabular orientation [13]. Whilst the effects of LSTV on the spine and spino-pelvic complex are described extensively, there is a lack of data in the literature on whether the change in pelvic anatomy due to LSTV is also accompanied by a change in acetabular orientation. The acetabular orientation is defined by the acetabular inclination and the acetabular anteversion [14]. Besides defining the orientation of the native acetabulum, acetabular orientation needs to be assessed for cup placement in a total hip arthroplasty (THA) in the supine position [15,16]. Assessing acetabular orientation relative to the table plane in the supine patient may result in a misjudgment and cup misplacement due to a pelvic tilt [17,18,19]. Therefore, these measurements in the supine position against the table plane are defined as a functional acetabular orientation based on the dependence of the pelvic tilt, whereas the measurement against a plane including the anterior superior iliac spine and the pubic tubercle, defined as the anterior pelvic plane (APP), is not affected by the supine position and pelvic tilting [17,20]. 

Therefore, with this study we aim to examine the influence of LSTV on the pelvic version and the functional and anatomical acetabular orientation. 

## 2. Materials and Methods

We performed a retrospective cross-sectional matched-pair analysis approved by the institutional ethics committee (EA1/300/19) using computed tomography (CT) scans of the lumbar spine and pelvis. No informed consent was required, due to retrospective study design. The study was reported according to the guidelines of the STROBE statement. Reasons for performing a CT on the patients included: tumor-staging, intra-abdominal hemorrhage, infectious focus search, and trauma. CT scans were performed between 2016 and 2019. Exclusion criteria were primary bone tumors or bone metastases, spondylodesis, rheumatic diseases, pelvic osteotomies, fractures of the pelvis or spine, or incomplete image data. CT-scans not showing the entire lumbar spine, including the last thoracic vertebra, the pelvis, and the femur up to the trochanter major, were defined as incomplete images. Out of the 819 patients enrolled, 53 had LSTV. Three of the patients with LSTV had to be excluded due to inconsistent image data, resulting in a lack of proper 3D reconstructability. Fifty patients with LSTV met the inclusion criteria and were matched by age and sex using the propensity-score matching, with a matching factor of 0.005 against 50 patients without LSTV, resulting in a total cohort of 100 patients that were analyzed for acetabular alignment.

### 2.1. Image Assessment

CT scans were performed by either an 80-row or a 320-row CT scanner (Canon Aquillon Prime and Canon Aquillon One Vision, Canon Medical Systems, Otawara, Japan) and reconstructed in an isometric volume with 1.0 mm slice thickness in a medium soft-tissue kernel with beam-hardening compensation. LSTV were classified by a radiological consultant (K.Z.) with specialization in musculoskeletal radiology according to the Castellvi classification (Table 1) [4]. An orthopedic surgeon (N.T.) with experience in radiologic measurements performed the image reconstruction and measurement, and was therefore, trained by an orthopedic attending surgeon (M.P.).

### 2.2. Measurements

All measurements were performed using Amira for Life and Biomedical Sciences (Thermo Fisher Scientific Materials and Structural Analysis c/o Zuse-Institut, Berlin, Germany). Image segmentation between bone and soft tissue was conducted using threshold segmentation with a lower limit of 200 Hounsfield units for bone. For better exposure of the acetabulum, the femur bone and femoral head were manually removed. The remaining osseous structures were then 3D reconstructed.

**Figure 1:**
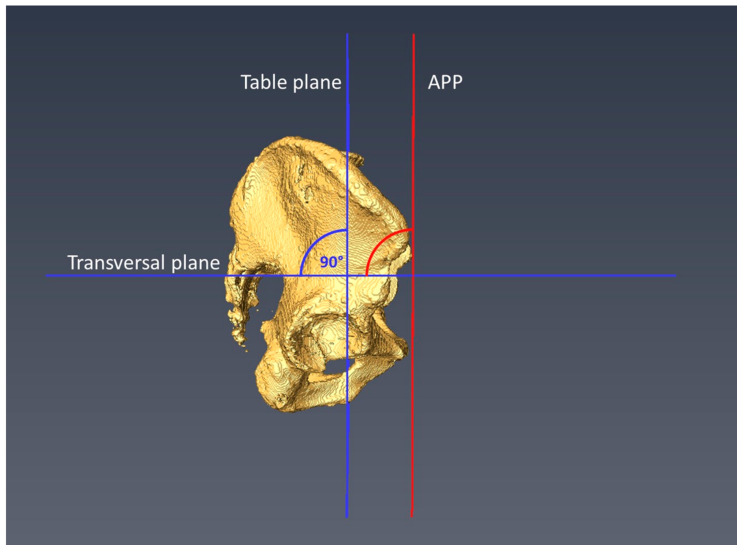
The measurement of the pelvic tilt in relation to the APP (anterior pelvic plane) is depicted.

Pelvic tilt was determined by the angle of the APP relative to the table plane in the sagittal plane. A plane containing the spinae iliacae anteriores superiores and the two pubic tubercles defined the APP, as shown in Figure 1. Pelvic tilt was calculated as the measured angle of the APP against the transversal plane followed by a subtraction of 90°.

**Figure 2:**
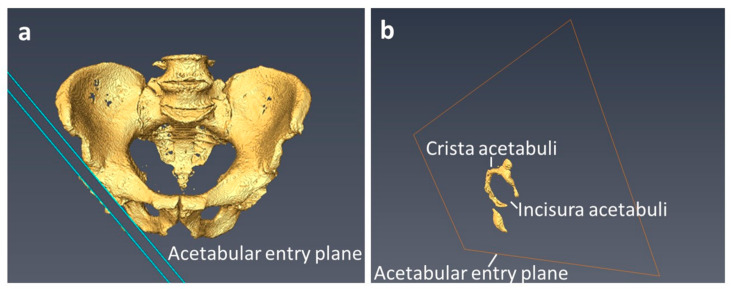
Acetabular entry plane: (**a**) Visualizes the acetabular entry plane with the bony irregularities of the crista acetabuli slightly laterally displaced for better illustration. The acetabular entry plane defines the acetabular orientation and reproduces the crista acetabuli as accurately as possible. (**b**) Shows the crista acetabuli in relation to the acetabular entry plane, which has been moved toward the acetabular fossa for better visualization.

Pelvic incidence (PI) was measured as the angle between the line from a bicoxofemoral axis to the midpoint S1 upper endplate and a line perpendicular to the S1 endplate containing the midpoint of S1 upper endplate. The acetabular orientation was determined for both hips. For the determination of the acetabular orientation, a plane was defined according to Higgins et al., which depicted the osseous acetabular entry plane as accurately as possible, except for the incisura acetabuli, as shown in Figure 2 [17,20].

**Figure 3:**
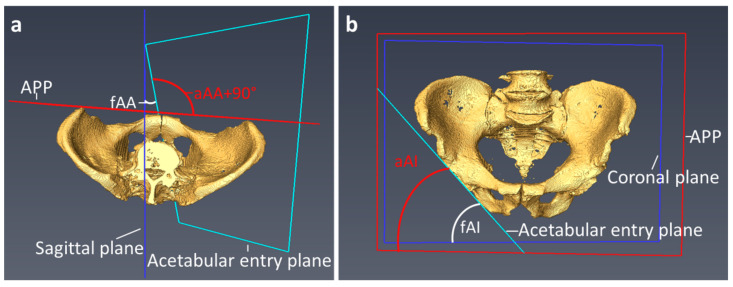
Measurement of the functional and anatomical acetabular orientation: (**a**) Shows the measurement of acetabular anteversion. (**b**) Demonstrates the measurement of acetabular inclination. APP = Anterior pelvic plane, fAA = functional acetabular anteversion, aAA = anatomical acetabular anteversion, fAI = functional acetabular inclination, aAI = anatomical acetabular inclination.

The acetabular anteversion was measured through the angle of the acetabular entry plane and the table plane in the transversal plane for the determination of the functional acetabular anteversion (fAA). Anatomic acetabular anteversion (aAA) was determined by the angle between the acetabular entry plane and the APP in the transverse plane, as shown in Figure 3a. The functional anatomical anteversion (fAA) was measured by the angle between the sagittal plane and the acetabular entry plane in the transversal plane. The anatomic acetabular anteversion (aAA) results from the angle of the acetabular entry plane and the anterior pelvic plane (APP), from which 90° had to be subtracted to obtain the aAA.

The functional acetabular inclination (fAI) was determined by the angle between the acetabular entry plane and the longitudinal axis of the coronal plane. The anatomical acetabular inclination (aAI) was determined by the angle between the acetabular entry plane and the longitudinal axis of the APP, as shown in Figure 3b.

For the reduction of systematic bias due to an oblique supine position of the pelvis, the values obtained for each patient from the right and left hip of each patient were averaged.

### 2.3. Statistical Analysis

Statistical analyses were performed using SPSS Version 27 (IBM Corporation, New York, NY, USA). The Kolmogorov Smirnov test was used to test data for normal distribution. For the statistical analysis of paired parametric data, the paired T-test was used. For nonparametric-paired data, the Wilcoxon rank sum test was used. Spearman’s correlation coefficient was used for correlation analysis of nonparametric data, Pearson correlation coefficient for parametric data. The level of significance was set at *p* < 0.05 for all tests.

## 3. Results

### 3.1. Demographics

Castellvi grading for the included 50 patients with LSTV is given in Table 2. Demographics of the matched cohorts of 50 patients with LSTV and control group of 50 patients is given in Table 3.

### 3.2. Influence of the Pelvic Tilt on the Acetabular Orientation in the Entire Cohort

In the cohort of 100 patients, the fAI was measured in relation to the table plane, and was significantly lower when compared to the aAI (*p* < 0.001). For acetabular anteversion, no significant differences were found for the fAA compared to the aAA (*p* = 0.783) as presented in Table 4. However, functional parameters showed significant correlation with anatomical parameters for both acetabular inclination (*p* < 0.001, r = 0.975) and acetabular anteversion (*p* < 0.001, r = 0.985).

Patients with a greater anatomical acetabular inclination had a significantly higher pelvic tilt (*p* < 0.001, r = 0.363). The anatomical acetabular anteversion did not significantly correlate to pelvic tilt (*p* = 0.235 r = −0.120). However, the extent of the difference between the supine position (functional) and anatomical parameters showed a significant correlation with the extent of the pelvic tilt for inclination (*p* < 0.001, ρ = 0.536) as well as for anteversion (*p* = 0.046, ρ = 0.200).

### 3.3. Influence of Pelvic Incidence on Acetabular Orientation

Significant differences in PI between LSTV and the control group was observed (*p* < 0.001; LSTV 61.3 ± 10.7, control 50.7 ± 8.6). No correlation in patients with LSTV of PI to fAA (*p* = 0.323), aAA (*p* = 0.341), fAI (*p* = 0.507), aAI (*p* = 0.609) as well for the control group of PI to fAA (*p* = 0.412), aAA (*p* = 0.502), fAI (*p* = 0.261), aAI (*p* = 0.388) was observed.

### 3.4. Influence of LSTV on Acetabular Orientation

**Figure 4:**
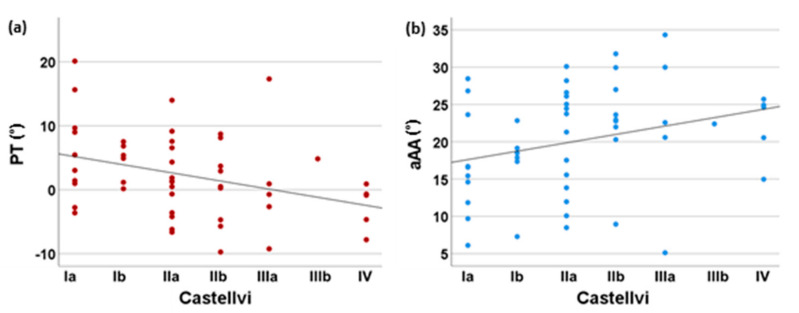
Influence of the Castellvi degree of LSTV on pelvic tilt and anatomical acetabular anteversion. In (**a**), the significant negative correlation between the degree of LSTV according to Castellvi and the pelvic tilt is given. (**b**) Depicts the significant positive correlation between anatomical acetabular anteversion and the degree of LSTV according to Castellvi. LSTV = Lumbosacral transitional vertebrae, PT = pelvic tilt, aAA = anatomical acetabular anteversion.

The degree of LSTV, according to Castellvi, was correlated with a significantly reduced pelvic tilt in our patient collective (*p* = 0.006, ρ = −0.387), as presented in Figure 4. Patients with an increasing Castellvi grading of LSTV showed a significantly larger fAA (*p* = 0.022, ρ = 0.324) and aAA (*p* = 0.031, ρ = 0.306). Regarding fAI (*p* = 0.493) or aAI (*p* = 0.850), the degree of LSTV had no significant influence. Apart from these observations, patients with LSTV did not differ significantly from the control group in terms of fAI (*p* = 0.765), aAI (*p* = 0.748), fAA (*p* = 0.266), aAA (*p* = 0.282) as presented in Table 5.

## 4. Discussion

To the authors’ knowledge, this is the first study to address the acetabular orientation in patients with LSTV. Even if no significant differences between LSTV and the control group regarding PT, as well as acetabular orientation, was detected, the degree of expression of LSTV correlated significant with acetabular anteversion. No correlation between pelvic geometry, defined by pelvic incidence and acetabular orientation was observed. Castellvi grading in patients with LSTV correlated negatively with pelvic tilt. Therefore, LSTV and Castellvi grading might be assessed on pre-operative X-rays prior to THA, and thus surgeons might consider adjusting cup positioning accordingly.

Our data for the anatomical mean acetabular anteversion of 19.4° and an acetabular inclination of 49.6° are in the range of those reported in the literature [17,20,21]. Cup misplacement in THA with increased or reduced anteversion or inclination may result in an increased risk of dislocation and implant wear, therefore, acetabular orientation needs to be assessed for THA [22]. The concept of a safe zone for the acetabular component was first described by Lewinnek et al.; they defined it with a range of 15 ± 10° anteversion and 40 ± 10° inclination to minimize the risk of dislocation [16]. Nevertheless, Esposito et al. demonstrated that 57% of their cups were in the alleged safe zone when dislocation occurred [23]. In accordance with this experience, Hevesi et al. redefined the safe zones, ranging from 27° to 47° of inclination and 18° to 38° of anteversion; however, dislocations were still reported with the adapted safe zones [24]. These results are not surprising, since these criteria were defined on two-dimensional radiographs and dislocations occur in motion. Due to the gait dependence of acetabular orientation, the discussion has moved away from the concept of a static safe zone towards a functional safe zone [25], which is reflected by the finding of Dandachli et al., who reported a decrease of 2.5° to 5° for every 5° of pelvic forward tilting [26]. This finding is supported by the significant correlations of pelvic tilt on acetabular inclination and the influence on acetabular anteversion in our results. Consistent with our results, the literature reported a significant influence of pelvic tilt on acetabular orientation, as well as on acetabular cup positioning in THA [18,26,27,28,29]. Therefore, Ross et al. and Maratt recommend a preoperative adjustment of THA planning to the pelvic tilt to reduce the risk for functional misplacement [18,27]. However, even in extreme pelvic movements, such as rising from sitting to standing, which is associated with differences in pelvic tilt of up to 20°, adequate coverage of the femoral head by acetabular cup must be ensured to avoid luxation [30]. The correlation between the pelvic tilt and LSTV were reported controversially [8,9,31]. Benlidayi et al. described reduced sacral tilt in 85 patients with LSTV [31]. Yokoyama et al. and Price et al. described elevated values for pelvic tilt in patients with an aberration of the count of lumbar vertebrae [8,9]. Whereas in our cohort, a higher degree of expression of LSTV correlated to a significantly reduced pelvic tilt, even if no significant differences between LSTV and control group were observed. These differences between both studies could possibly have resulted from the differences in assessment between the supine position in our study compared to standing position and the definition of pelvic tilt by deflection of the APP relative to the table plane. However, high interindividual differences in acetabular orientation were observed. Therefore, as a clinical implication, intraoperative referencing for cup positioning should be performed against the APP instead of the table plane, even in patients with LSTV, to avoid misplacement due to the influence of pelvic tilt, as well as the interindividual anatomical differences.

To the authors knowledge there is no literature examining the influence of LSTV on acetabular orientation. The degree of expression of LSTV correlated significantly with increased functional and anatomical acetabular anteversion. Whereas patients with LSTV and the control group did not differ significantly in acetabular orientation. This might possibly result from the distribution of patients with LSTV with predominantly low Castellvi grading in our cohort.

LSTV were associated with an increased pelvic incidence compared to control group, which significantly determined the pelvic geometry as presented by Haffer et al. [6]. In line with the literature in our study, no correlation between acetabular orientation and pelvic incidence for the control group was observed [32]. Furthermore, for patients with LSTV, no correlation between pelvic incidence and acetabular orientation was found.

Some limitations of the study must be mentioned. A selection bias for the assessment of acetabular orientation could have resulted from the predefined demographics of the control group by the LSTV cohort, as well as the central European patient’s cohort. We defined the acetabular entry plane according to the existing osseous structures, omitting the incisura acetabuli, and did not optimize for osseous appositions due to osteophytes or femoro-acetabular impingement. This corresponded most closely to the landmarks found intraoperatively for orientation during THA. In addition, statistical power was limited by lower case numbers, especially in higher-grade Castellvi groups and should, therefore, be interpreted as preliminary data. We did not determine the extent of the oblique pelvic position in the supine position. To reduce bias opportunities due to extreme values caused by the oblique lying, we averaged the measurements of both hips. The acetabular orientation was assessed in the supine position and thus, reflects the surgeon’s perspective intraoperatively, but cannot be unrestrictedly transferred to other body positions due to differences in pelvic tilt caused by spino-pelvic mobility in dynamic processes, such as walking or standing to sitting.

## 5. Conclusions

Concluding the study results, we detected that the degree of expression of LSTV correlated significantly with acetabular anteversion, even if no significant differences between LSTV and the control group in PT and acetabular orientation was observed. Besides high interindividual variability, Castellvi grading in patients with LSTV correlated negatively with pelvic tilt. The increased acetabular anteversion related to LSTV with higher Castellvi degree might be considered when planning or performing THA, even if data should be interpreted as preliminary data, due to small sample size in groups with higher Castellvi degrees. The pelvic tilt had a significant effect on the assessment of acetabular orientation in supine patients, even if the effect was only about 1°, which could be hard to account for by performing THA. However, for optimizing cup placement, individual anatomy including LSTV should be considered and acetabular orientation should be assessed based on APP, which the surgeon can evaluate intraoperatively by palpating the bony structures of the pelvis.

## Figures and Tables

**Table 1:** Classification according to Castellvi.

Castellvi Type	Description	Patients Example
I	Ia: Unilateral dysplastic transversal process > 19 mm Ib: Bilateral dysplastic transversal process > 19 mm	Ib 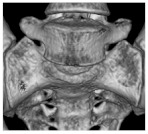
II	IIa: Unilateral pseudarthrosis between transversal process and sacral bone IIb: Bilateral pseudarthrosis between transversal process and sacral bone	IIa 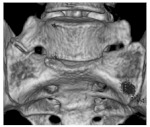
III	IIIa: Unilateral bony union between transversal process and sacral bone IIIb: Bilateral bony union between transversal process and sacral bone	IIIb 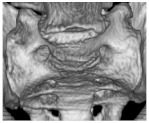
IV	Unilateral bony union contralateral pseudarthrosis between transversal process and sacral bone	IV 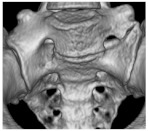

**Table 2:** Classification of patients with LSTV, according to the Castellvi.

Castellvi	I	II	III	IV
Patients (*n*)	16 (32%)	23 (46%)	6 (12%)	5 (10%)

LSTV = Lumbosacral transitional vertebrae.

**Table 3:** Patient’s characteristics.

	Control Group	LSTV
Age (years) mean (SD)	51.9 (20.1)	52.0 (17.6)
Sex		
Female (*n*)	23	23
Male (*n*)	27	27
5 Lumbar vertebrae (*n*)	50	32
6 Lumbar vertebrae (*n*)	0	11
4 Lumbar vertebrae (*n*)	0	7

LSTV = Lumbosacral transitional vertebrae, SD = standard deviation.

**Table 4:** Functional and anatomical acetabular anteversion and inclination.

	Anatomical (±SD)	Functional (±SD)	*p*-Value
Anteversion	19.4 (7.5)	19.4 (7.5)	0.783
Inclination	49.6 (7.4)	48.8 (6.8)	**<0.001**

Significant differences are marked in bold. SD = standard deviation.

**Table 5:** Acetabular orientation in patients with LSTV and the control group.

	LSTV (*n* = 50) (±SD)	Control (*n* = 50) (±SD)	*p*-Value
Pelvic tilt (°)	2.2 (6.6)	1.4 (7.8)	0.553
fAA (°)	20.2 (7.0)	18.5 (8.0)	0.266
aAA (°)	20.2 (7.2)	18.6 (7.7)	0.282
fAI (°)	49.1 (6.7)	48.6 (7.1)	0.765
aAI (°)	49.9 (7.2)	49.3 (7.7)	0.748

This table presents the mean values and standard deviations (SD) of the acetabular orientation of the LSTV and the matched control group. LSTV = Lumbosacral transitional vertebrae.

## Data Availability

All data is reported in the article.
